# Rethinking gut microbiome residency and the *Enterobacteriaceae* in healthy human adults

**DOI:** 10.1038/s41396-019-0435-7

**Published:** 2019-05-14

**Authors:** Jonathan N. V. Martinson, Nicholas V. Pinkham, Garrett W. Peters, Hanbyul Cho, Jeremy Heng, Mychiel Rauch, Susan C. Broadaway, Seth T. Walk

**Affiliations:** Department of Microbiology and Immunology, Montana, State University, Bozeman, MT USA

**Keywords:** Microbiome, Microbial ecology, Population dynamics, Population genetics

## Abstract

Longitudinal human gut microbiome datasets generated using community-level, sequence-based approaches often report a sub-set of long-lived “resident” taxa that rarely, if ever, are lost. This result contrasts with population-level turnover of resident clones on the order of months to years. We hypothesized that the disconnect between these results is due to a relative lack of simultaneous discrimination of the human gut microbiome at both the community and population-levels. Here, we present results of a small, longitudinal cohort study (*n* = 8 participants) of healthy human adults that identifies static and dynamic members of the gut microbiome at the clone level based on cultivation/genetic discrimination and at the operational taxonomic unit/amplified sequence variant levels based on 16S rRNA sequencing. We provide evidence that there is little “stability” within resident clonal populations of the common gut microbiome bacterial family, *Enterobacteriaceae*. Given that clones can vary substantially in genome content and that evolutionary processes operate on the population level, these results question the biological relevance of apparent stability at higher taxonomic levels.

## Introduction

Fundamental aspects of the adult human gut ecosystem remain understudied, including why and/or how some microbes colonize and persist while others do not. Estimates of residency (the length of time an organism occupies a niche) in the gut are scarce even for bacteria easily isolated on selective and/or differential media. The few cultivation-based, longitudinal studies of human adults suggest that strain-level turnover in the gut is common over relatively short time scales (i.e., months) [1–4]. Given the variability in genome content between bacterial strains of the same species [5], this variation could potentiate important functional dynamics. In contrast, the few studies that examined longitudinal gut microbiome dynamics using 16S rRNA [6–9] and shotgun metagenomic sequencing [10] suggest that a significant sub-set of the microbiome is highly stable, remaining present perhaps for an individual’s entire adult life [11, 12].

At least some of the above discrepancy is likely due to the scarcity of published studies. However, the discriminatory power of sequencing-based versus cultivation-based approaches is also likely to lead to different conclusions. For example, 16S sequencing is notoriously inconsistent or, depending on one’s view, inaccurate at identifying bacterial species [13], and is by design blind to population-level dynamics. Furthermore, sequencing-based studies rarely discuss or quantify lower limits of detection (e.g., concentrations down to ~10^6^ CFU/gram feces for 16S sequencing [14]), making it difficult to understand sampling effort. Shotgun metagenomic sequencing can also be limited by genome coverage (i.e., the amount of the genome needed to accurately differentiate between strains), which limits accurate strain-level identification. It seems at least possible that -omic sequencing approaches skew important attributes of microbial populations in the gut and in other environments. In this study, we sought to simultaneously quantify and compare community and population-level diversity to better clarify which elements of the gut microbiome are static versus dynamic.

*Enterobacteriaceae* is the most taxonomically diverse bacterial family [15] recognized by the International Committee on Systematic Bacteriology [16]. Most research on *Enterobacteriaceae* in humans has narrowly focused on the epidemiology, pathogenesis, virulence, and/or antibiotic resistance of pathogenic strains. Similarly, despite the ability to cultivate all (or nearly all) of the currently named *Enterobacteriaceae* species on a single selective, differential medium (MacConkey agar), there are few longitudinal studies from healthy human adults. As a result, it is difficult to extrapolate fundamental aspects of non-pathogenic *Enterobacteriaceae* ecology (e.g., prevalence and diversity) from studies of pathogenic and/or antibiotic resistant strains from hospitalized patients [17].

For this study, we collected semi-weekly stool samples from a cohort of eight healthy human adults and quantified the abundance and distribution of resident and transient operational taxonomic units (OTUs) and amplified sequence variants (ASVs) using 16S rRNA sequencing. From the same samples, we generated a large collection of *Enterobacteriaceae* isolates (*n* = 32,470) from MacConkey agar plates and identified residents at the individual clone level. The dynamics observed with cultured *Enterobacteriaceae* isolates are at odds with community-level stability and suggest that the adult human gut microbiome is not as stable as previously reported. Finally, with respect to longitudinal studies of healthy human adults, this is the first study to report statistical evidence that certain *Enterobacteriaceae* clones are more likely to be resident compared to others.

## Methods

### Sample collection and processing

This study was approved by the institutional review board of Montana State University. All participants were enrolled with informed consent, and a total of 9 volunteers (3 female, 6 male) were recruited, ranging in age from 25 to 40 years of age. No information beyond sex and age range was recorded, as correlating host factors with temporal dynamics was not the focus of this study. Although not asked directly, none of the participants co-habitated to our knowledge. Stool samples were requested once every 2 weeks from March 2016 to January 2018 (Table 1). Samples (~5 g of stool) were self-collected using disposable (plastic) commodes (Fisherbrand^TM^ Commode Specimen Collection System, Thermo Fisher Scientific, Inc.) into sterile 50 mL conical screw-cap tubes using a sterile tongue depressor. If samples could not be processed immediately, they were refrigerated at 4 °C for no longer than 2 hours prior to processing. Samples were processed inside an anaerobic chamber (Coy Laboratory Products, Inc., Grass Lake, MI, USA) to help preserve anaerobic bacteria for future investigations. Each sample was mixed by shaking with ~30 mL of pre-reduced, sterile phosphate buffered saline. Aliquots (1 mL) were then mixed with 0.2 mL of 80% glycerol in sterile cryogenic vials containing gaskets (Neptune® 1.5 mL CryoTube, VWR International part of Avantor®, Radnor, PA, USA) at a final glycerol concentration of 15%. In parallel, additional aliquots (0.2 mL of stool) were made directly into DNeasy Power Soil DNA extraction tubes (Qiagen, Hilden, Germany) for bulk DNA extraction prior to 16S rRNA sequencing (see below). All aliquots were immediately frozen at −80 °C.

### Sampling and characterization of resident clones

Fecal slurries were thawed to room temperature, serially diluted in sterile PBS, and plated onto MacConkey agar plates, followed by overnight incubation at 37 °C. A target of 95 single colonies per sample were picked from plates using sterile toothpicks and transferred into lysogeny broth (LB) in a 96 deep-well format. One well served as an uninoculated control. If lactose fermenting and non-fermenting colonies were present, each type was picked at roughly the ratio present on the plate. In addition, care was taken to select colonies that differed in morphology, such that rare, odd-looking colonies were present in downstream characterizations. This approach was used to maximize the probability of observing diverse *Enterobacteriaceae* species as opposed to precisely estimating true abundances. LB cultures were incubated overnight (37 °C), and freezer stocks were made in 15% glycerol and immediately frozen at −80 °C.

Diluted (1:10) cultures were used for PCR-based genotyping; first using the *E. coli* phylogrouping algorithm of Clermont et al. [18] (half-reaction volumes were used with 1.6 µL of diluted culture; Supplementary Fig. 1). Negative PCRs were repeated twice. If the PCR failed a second time, the isolate was presumed to belong to a species other than *E. coli*. Residents were defined as described in the Results section. Resident clones were identified as having identical GTG5 rep-PCR fingerprints [19] (Supplementary Fig. 2). Non-*E. coli* residents were characterized biochemically (API 20E test strips) to identify their likely species designation. In one case, a cryptic *Escherichia* clade was confirmed by multilocus sequence typing (Sanger sequencing) following a published protocol [20]. Resident clones belonging to phylogroups A and B2.3 were clonotyped using methods described by Tchesnokova et al. [21].

### 16S rRNA sequencing and analysis

DNA was extracted from fecal slurries (DNeasy PowerSoil kit, QIAGEN) and the V4 region of 16S rRNA encoding gene was amplified and sequenced as described [22] at the University of Michigan Center for Microbial Systems using Illumina MiSeq 2 × 250 bp paired-end sequencing. Reads were generated on two separate sequencing runs in February and June of 2018.

Raw reads were processed using mothur v.1.39.5 [23]. Low-quality reads were removed following the mothur SOP [22] (accessed on May 7, 2017). Briefly, forward and reverse reads were assembled into 253 bp long contigs. Contigs containing ambiguous bases or homopolymers >8 bp were discarded. Identical sequences were combined, and the remaining sequences were aligned against the SILVA database (version 128) trimmed to the V4 region. A pre-cluster step was used to combine rare contigs with more abundant members in the dataset if they differed at 3 or fewer nucleotide sites. Chimeras were identified and removed in mothur using UCHIME [24]. Singletons and doubletons were removed prior to clustering. Sequences sharing 97% similarity were binned into Operational Taxonomic Units (OTUs) using VSEARCH [25], and classified within mothur using the Ribosomal Database Project’s Bayesian classifier (training set 10) [26]. OTUs represented by less than 100 sequences in the dataset were removed to guard against spurious reads. In parallel, quality filtered reads were assembled into amplicon sequencing variants (ASVs) using the DADA2 v1.10.1 pipeline [27]. Trimming and chimera removal were done following the DADA2 Pipeline Tutorial (1.8), and ASVs were classified using the SILVA database (version 128). All OTUs/ASVs that classified to mitochondria, chloroplast, Eukaryota, or remained unclassified at the domain level were removed. Of the original 9 350 936 reads, 7 055 736 and 7 109 813 were assigned to OTUs and ASVs, respectively.

Reads from each sample were rarefied to 10,000 and all downstream analyses were conducted using R version 3.5.1 as follows: PERMANOVA was conducted using the Adonis function in vegan 2.5-3 and 1 000 permutations [28], partitioning around mediods (PAM) clustering was conducted using cluster 2.0.7-1 [29], and ecological diversity estimates and ordinations were generated with Labdsv 1.8-0 [30], and custom R and Python scripts [30, 31, 32].

## Results

### Study participants and stool samples

Nine healthy volunteers between 24 and 40 years of age (3 females, 6 males) were enrolled with informed consent. No other information was collected (height, weight, BMI, etc.), but all were well enough to participate and presumed to represented healthy adults in this geographic location. Only a single enrollee did not participate beyond three sampling dates and was excluded. From the remaining eight, a total of 392 stool samples were collected, averaging 49 samples per individual (range = 25–97), spanning an average of 512 days (range = 245–849 days), and a median of 8 days apart (Supplementary Fig. 3). All samples were plated directly onto MacConkey agar plates for evaluation of population-level dynamics, and a sub-set of samples (n = 324) were used to evaluate community-level dynamics by 16S rRNA sequencing.

### 16S rRNA sequencing reveals similar microbiomes within participants over time

**Fig. 1:**
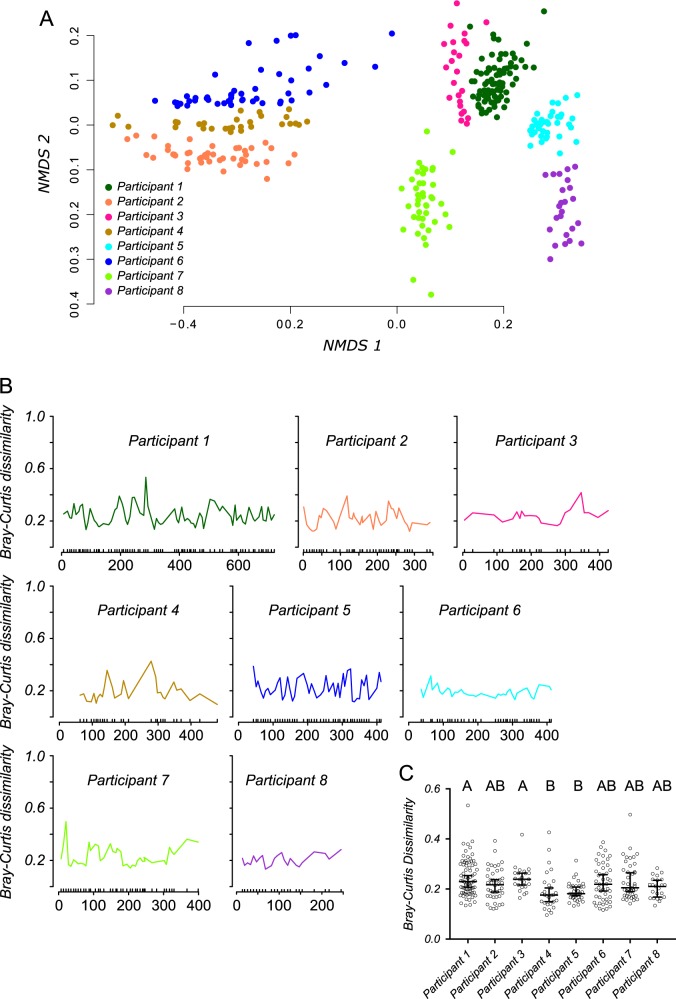
16S rRNA sequencing-based OTU analysis. Non-metric multidimensional scaling (NMDS) of microbiomes from different participants (**a**). Each sample is represented by a dot and colors correspond to participants. Bray-Curtis dissimilarity between consecutive samples was plotted through time (**b**). Differences in Bray-Curtis dissimilarities shown in panel B were tested for significant participant-wise differences (**c**; Kruskal-Wallis test; *p* < 0.0001; error bars represent median and 95% confidence limits). Significant differences (*p* < 0.05) following correction for multiple comparisons (*p* < 0.05; Dunn’s test) between groups are summarized above plots by letters. Participants that share letters were not significantly different

Stool samples from eight participants were sequenced using dual-index barcoding and paired-end Illumina MiSeq sequencing (V4 region), resulting in 293 OTUs among all eight participants. Non-metric multidimensional scaling (NMDS) grouped samples according to participant, and PERMANOVA (*f* = 176.46, *p* < 0.001) supported statistically different groups (Fig. 1a). PAM clustering was used to identify the most statistically supported number of clusters by evaluating the average silhouette width for 2 to 100 clusters. Seven clusters had the highest support; six of which were comprised of participants 1, 3, 5, 6, 7, 8 and a seventh comprised of participants 2 and 4 with highly similar microbiomes (Supplementary Fig 4). Beta diversity (Bray-Curtis dissimilarity) within participant 2 or 4 was statistically lower than between these participants (Wilcoxon rank sum test *p*-value < 2.2e-16). However, 150 of the 1 247 (12%) between-participant comparisons yielded lower beta diversity than the median within-participant diversity, highlighting the high degree of similarity between these microbiomes.

The change in beta diversity from one sampling date to the next was typically small for all participants (Fig. 1b and error bars in Fig. 1c), and some participants (4 and 5) displayed significantly lower overall diversity compared to others (Fig. 1c). We also evaluated whether beta diversity increased with time and a significant positive correlation (Pearson correlation) was found for all but Participant 2 (Supplementary Fig. 5). However, both the correlation coefficients (range = 0.056–0.377) and the effect size of beta diversity change (range = 0.04–0.13) were small, suggesting that microbiomes sampled close in time were nearly as similar as those sampled over long periods of time.

ASV-based analysis identified 453 ASVs among participants and overall, NMDS (Supplementary Fig. 6), PERMANOVA (*f* = 207.69, *p* < 0.001), and PAM clustering (Supplementary Fig. 4) were highly similar to OTU-based results. Interestingly, Procrustes analysis of OTU and ASV-based ordinations (Supplementary Fig. 7) revealed very different sum of squared distances (SSDs) across participants (Participant 2 had 20-fold greater SSDs compared to Participant 7), suggesting that ASVs and OTUs produce nearly identical results for some but not all participants. With respect to PAM clustering, larger mean silhouette widths were generated using ASVs, resulting in well-supported clusters corresponding to all eight participants. Because more ASVs were identified compared to OTUs, estimates of alpha (Inverse Simpson) and beta diversity were expectedly lower with OTUs. However, ASV and OTU estimates of both alpha and beta were highly correlated (Pearson correlation; coefficients for alpha range = 0.9097–0.9860; coefficients for beta range = 0.8638–0.9903) in each participant (Supplementary Fig. 8 and 9).

### OTU/ASV residency varies significantly

Residency was defined based on human gut transit time. We reasoned that bacteria entering the gut would be lost at the rate of gut transit *unless* they were able to become established and colonize. Based on a previously published gut transit time (0.7–4 days) for a dietary residue in healthy human adults [33], we set a threshold of 14 days, meaning that to be considered a resident, OTUs/ASVs had to overcome at least 3 times the maximum transit time. We also allowed for gaps, such that an OTU/ASV could be absent for no more than 30 days before assuming it was lost. If the OTU/ASV reappeared, it would have to again fulfill a 14-day threshold before being considered a resident again. Thus, our definition allowed for both a binary (yes/no) evaluation of residency and quantification of residence time (days). Finally, because not every stool sample produced by each participant was collected, we included flexibility into the way that time was estimated such that a minimum residency period (Min) was the difference between the first and last dates that an OTU/ASV was observed; the maximum (Max) residency period was the difference between the date prior to the first observation and the date immediately following last observation; and the average (Ave) residency period was the difference between the date of the first observation and the date immediately following the last observation (Supplementary Fig. 10).

The most stable sub-set of the microbiome was those OTUs/ASVs that were resident for the entire study period (Always Resident), corresponding to an average of 34% (Min), 39% (Ave), or 48% (Max) of OTUs and 27% (Min), 32% (Ave), or 41% (Max) of ASVs observed within participants (Table 1). To understand whether resident OTUs in one participant were residents in other participants (i.e., a “core resident microbiome”), we plotted the number of OTUs that were always resident in at least “x” number of participants (from 1 to 8, Supplementary Fig. 11). In such plots, lines would plateau at a non-zero threshold if at least some of OTUs were always observed. Exponential decay equations fit very well to curves according to each residency period estimation method (R square = 0.99 for each), but none of the modeled plateaus (i.e., predicted number of OTUs if an infinite number of participants were considered) significantly differed from 0 (*p* = 0.1976, 0.2502, 0.4157 for Min, Ave, and Max, respectively). Thus, there was little support for a “core resident microbiome”.

**Table 1:** Summary of samples, sampling days, number of OTUs, and residence time according to operationally defined estimates of time (see Methods)

				Minimum			Average			Maximum		
Participant	Samples	Days	OTUs	Resident (%)	Transient (%)	Always resident (%)	Resident (%)	Transient (%)	Always resident (%)	Resident (%)	Transient (%)	Always resident (%)
1	76	722	220	170 (77)	50 (23)	63 (29)	196 (89)	24 (11)	76 (35)	215 (98)	5 (2)	92 (42)
2	43	342	244	181 (74)	63 (26)	90 (37)	206 (84)	38 (16)	99 (41)	223 (91)	21 (9)	105 (43)
3	24	425	189	151 (80)	38 (20)	65 (34)	179 (95)	10 (5)	80 (42)	185 (98)	4 (2)	95 (50)
4	29	480	186	145 (78)	41 (22)	51 (27)	163 (88)	23 (12)	57 (31)	178 (96)	8 (4)	97 (52)
5	38	412	179	137 (77)	42 (23)	62 (35)	158 (88)	21 (12)	77 (43)	177 (99)	2 (1)	92 (51)
6	48	412	181	111 (61)	70 (39)	56 (31)	125 (69)	56 (31)	63 (35)	145 (80)	36 (20)	82 (45)
7	42	399	242	196 (81)	46 (19)	80 (33)	213 (88)	29 (12)	90 (37)	231 (95)	11 (5)	107 (44)
8	24	245	181	142 (78)	39 (22)	83 (46)	158 (87)	23 (13)	94 (52)	171 (94)	10 (6)	106 (59)
Average	41	430	203	154 (76)	49 (24)	69 (34)	175 (86)	28 (14)	80 (39)	191 (94)	12 (6)	97 (48)

**Fig. 2:**
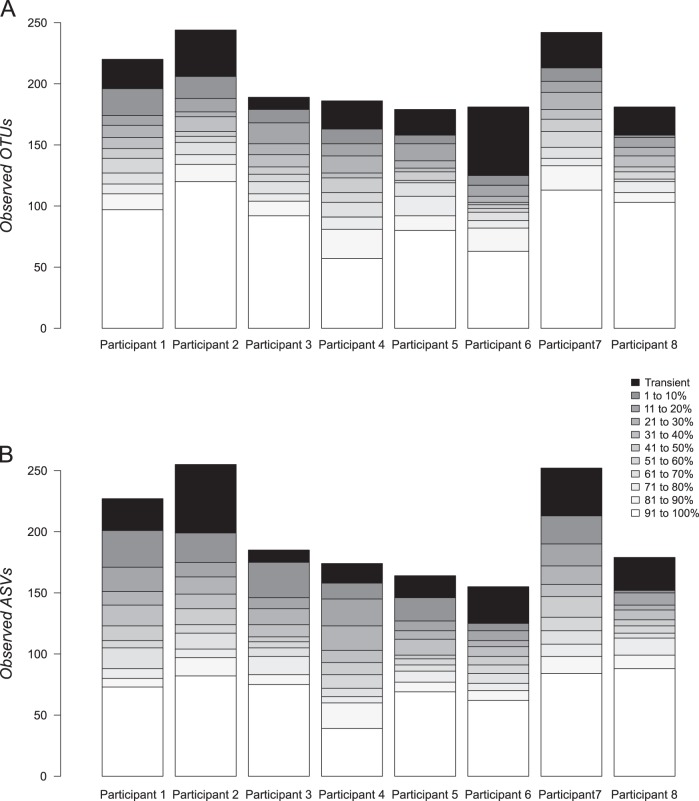
Stacked bar charts of microbiome residency using the average estimate. The number of observed OTUs (**a**) and ASVs (**b**) are shown with respect to percentile rank according to the percentage of time present in each participant using the average residency estimate. Black stacks in each bar correspond to transient OTUs and ASVs (i.e., never resident)

We next generated stacked bar charts of OTUs and ASVs according to percentile residency rank (Fig. 2, Supplementary Fig. 12). These plots showed that significant proportions of the microbiome were rarely resident and only an average of 55% (Min), 64% (Ave), or 72% (Max) of OTUs and 46% (Min), 55% (Ave), or 65% (Max) of ASVs were resident in participants for more than half (>50%) of the study period. Collectively, these results suggest that while the microbiome of participants maintained its uniqueness over time (i.e., NMDS), there appeared to be substantial flux in membership.

### Culture-based evaluation of *Enterobacteriaceae* in the healthy human adult gut

A total of 32 470 single colonies were picked from MacConkey agar plates from the same stool samples (*n* = 324) used for 16S sequencing and the additional 68 stool samples that were available (total = 392). An average of 84 colonies per stool sample per participant (SD = 4.7) were characterized by PCR-based assays, and the clear majority (*n* = 28 156, 87%) belonged to one of ten *E. coli* sensu stricto phylogroups. The percentage of non-*E. coli* varied between participants from <1 to 37% (mean = 13.27%, SD = 13.28%). Since all members of the *Enterobacteriaceae* known to colonize the human gut should be cultivable on MacConkey agar and since we attempted to isolate all colony morphology types, these results suggest that *E. coli* is by far the most dominant *Enterobacteriaceae* in healthy humans. Interestingly, a significant number of *E. coli* (*n* = 7699, 27%) did not appear to ferment lactose when grown on MacConkey agar, suggesting that this commonly used biochemical marker of *E. coli sensu stricto* requires re-evaluation.

### Enterobacteriaceae residency

**Fig. 3:**
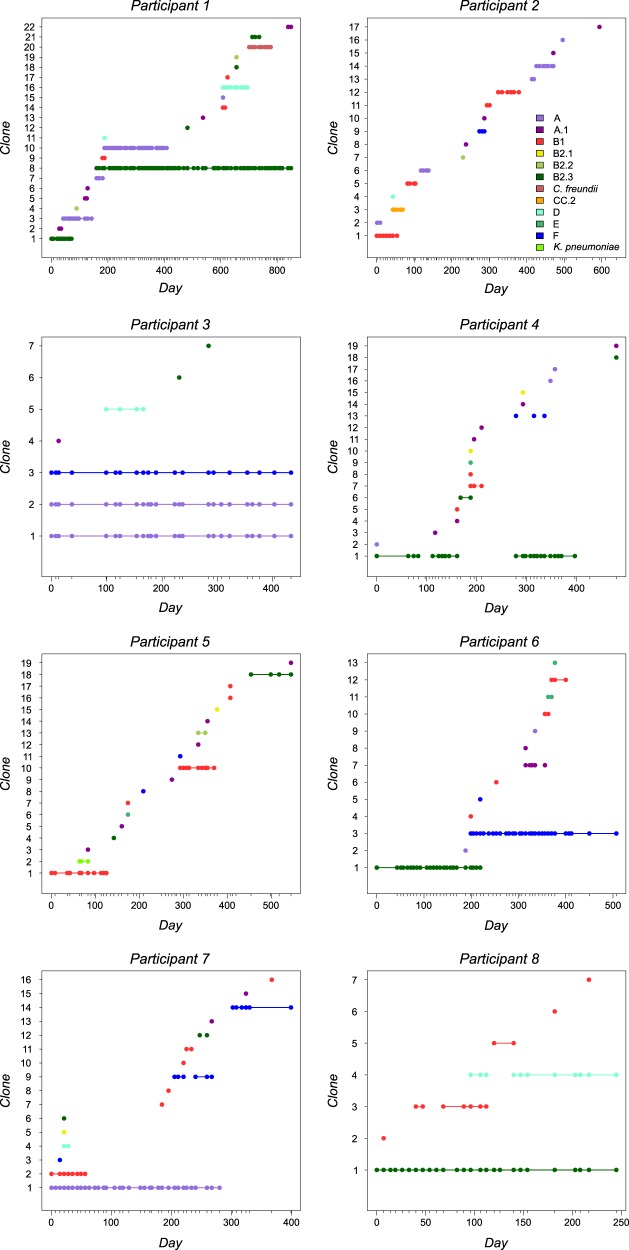
Presence of *Enterobacteriaceae* clones. Unique *Enterobacteriaceae* clones defined by PCR-based discriminatory assays (see Methods) where plotted according the day(s) observed. Day 0 in each panel corresponds to the first stool sample collected from both participants and ticks along the x-axis represent samples. Clones are ordered by order of appearance along the y-axis and colored according to either their phylogroup membership (*E. coli*) or species as defined by biochemical testing. The legend shown for Participant 2 is the same for all panels. No clones were shared between any participant (e.g., “Clone 1” in Participant 1 is not the same as “Clone 1” for any other participant)

GTG5 rep-PCR was used to identify distinct clonal lineages that met our residency definition and the presence of individual clones through time was plotted for each participant (Fig. 3). We also plotted the relative abundance of phylogroups within participants (Supplementary Fig. 13) and while we believe these plots reasonably represent true abundances, it should be noted that colonies were not necessarily selected at random (see Methods) and so some rarer members are likely overrepresented. It is also important to note that not all 32 470 isolates were fingerprinted to identify residents. Instead, we first identified potential residents using a step-wise algorithm (Supplementary Fig. 2), which allowed us to narrow down the number of isolates to a reasonable sub-set for rep-PCR. Some transients were identified using the combination of phylogrouping PCR results and residence time (≤14 days) and subsequently confirmed by rep-PCR. We did not fingerprint all transient isolates or try to identify all transient clones. For simplicity, we assumed that *E. coli* transients belonging the same phylogroup in the same sample also belonged to the same clone, which is a highly conservative assumption. Likewise, we did not attempt to identify all transient non-*E. coli Enterobacteriaceae* clones. Instead, we conducted rep-PCR on all non-*E. coli Enterobacteriaceae* from three of the eight participants (*n* = 116) and a reasonable random sub-set (22–68% of isolates) in the remaining participants (Supplementary Table 1). In total, 967 of the 3164 (31%) potentially resident non-*E. coli Enterobacteriaceae* were evaluated. Again, this approach provided an accurate estimate of the number of resident clones and a conservative estimate of the number of transient clones.

**Fig. 4:**
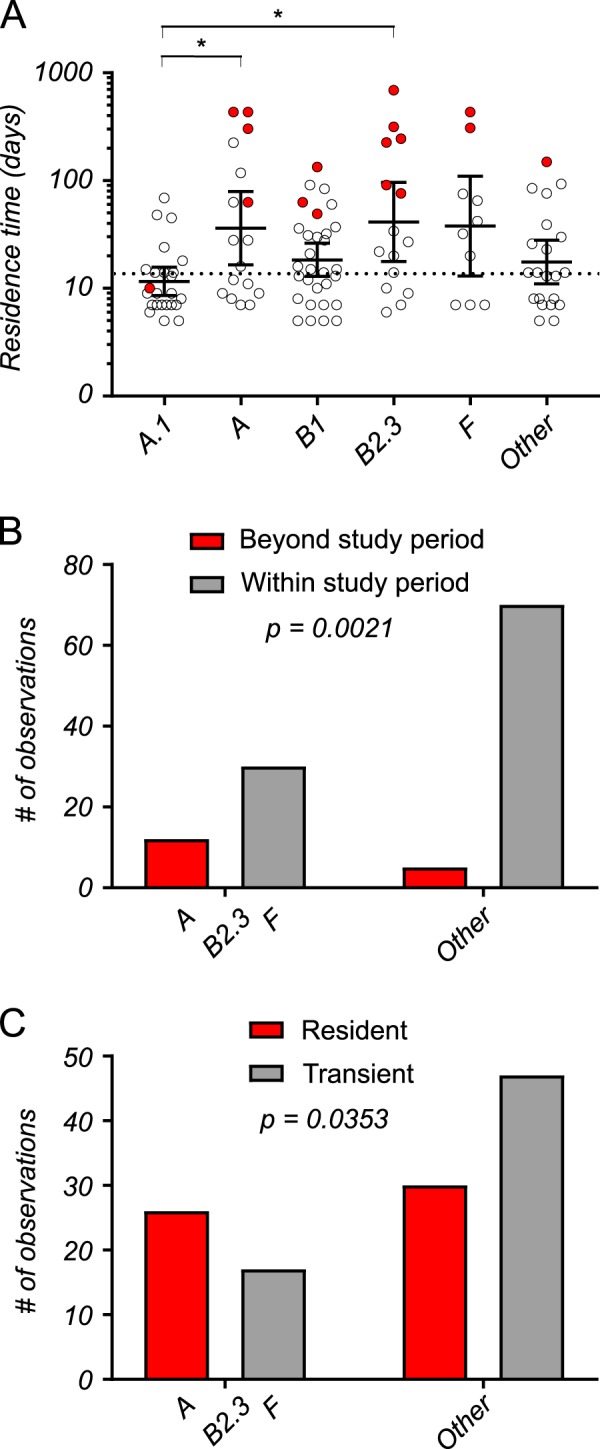
*Enterobacteriaceae* clone residency using the average estimate. All *Enterobacteriaceae* clones were plotted according to their log 10 transformed residence time in days (**a**). Clones were grouped according their phylogroup (*E. coli*) or into an “Other” category if ≤6 representative isolates were observed (Other = B2.1, *n* = 4; B2.2, *n* = 4; D, *n* = 6; E, *n* = 4; Cryptic *Escherichia* clade IV, *n* = 1; *C. freundii*, *n* = 1; *K. pneumoniae*, *n* = 1). Groups had significantly different residence time (ANOVA, F = 3.729, *p* = 0.0037) and following correction for multiple comparisons (Holm-Sidak’s test), groups A and B2.3 resided significantly longer than A.1 (*p* = 0.0338, *p* = 0.0171, respectively). Red dots identify clones that were present at the outset of the study, throughout the entire study period, or at the end of the study. These clones were presumed to have colonized participants beyond the study period. Contingency table analysis (Chi-square test) was used to test whether significantly more clones were observed beyond the study period in some groups versus others (**b**) or whether significantly more resident clones were present (**c**) among the longest lived phylogroups (A, B2.3, and F) compared to other groups. Both comparisons were significant and *p*-values are shown in each panel

A total of 120 *Enterobacteriaceae* clones were identified among all participants and 37 (31%) of these were resident. Note that because rep-PCR was not performed on all isolates, the number of transient clones was likely to be somewhat greater than 86. Of the 37 resident clones, only three were non-*E. coli*, and each belonged to a different “species” (*Citrobacter freundii*, *Klebsiella pneumoniae*, and cryptic *Escherichia* clade IV), suggesting that with respect to residency, *E. coli* was again the dominant species. Within *E. coli sensu stricto*, all phylogroups were observed with the single exception of phylogroup C. The most commonly observed phylogroup was B1 (*n* = 30 clones, 25%), followed by A.1 (*n* = 26, 22%), A (*n* = 17, 14%), B2.3 (*n* = 16, 13%), and F (*n* = 10, 8%). All other clones (B2.1, B2.2, D, and E) comprised ≤ 5% each and together accounted for only 15%. Residence time (log 10 transformed number of days observed) was significantly different between *Enterobacteriaceae* clones (Fig. 4a) using the Ave and Max estimates (ANOVA; Ave, *p* = 0.0037; Max, *p* = 0.0034) and approached significance using the Min estimate (ANOVA; *p* = 0.0735). Phylogroups A and B2.3 had the longest residence times, and following correction for multiple comparisons, both phylogroups resided significantly longer than phylogroup A.1 using both Ave (Fig. 4a) and Max (Supplementary Fig. 14) estimates. Phylogroup F clones also resided significantly longer than A.1 clones but using the Max estimate only (Supplementary Fig. 14). In addition to having the longest mean residence times, A, B2.3, and F clones were more often observed “beyond the study period” (Fig. 4b). Finally, A, B2.3, and F clones were identified as residents (as opposed to transients) more often (Fisher’s exact test, *p* = 0.024) using the Ave estimate (Fig. 4c). Given their greater overall residence times, likeliness to be present beyond the study period, and frequency as residents, these results suggest that *E. coli* belonging to A, B2.3, and F phylogroups are more likely to be human residents compared to all other members of the *Enterobacteriaceae*.

### 16S rRNA sequencing is not diagnostic for *Enterobacteriaceae* or *Escherichia*/*Shigella*

*Enterobacteriaceae* and *E. coli* were cultured from 308 and 289 of the 324 (95% and 89%, respectively) stool samples that were evaluated by 16S sequencing. Only two OTUs classified as *Enterobacteriaceae*, and one of these classified to the *Escherichia*/*Shigella* genus. Likewise, five ASVs classified as *Enterobacteriaceae* and one of these classified to the *Escherichia*/*Shigella* genus. By comparing OTU/ASV presence-absence to culture-based presence-absence across samples, we evaluated the diagnostic potential of 16S to identify *Enterobacteriaceae* or *Escherichia*/*Shigella* in stool samples. Counts for false positives, true positives, false negatives, and true negatives as well as positive and negative predictive values and accuracy were generated (Supplementary Table 3). Overall, 16S sequencing had excellent positive predictive value, regardless of OTU or ASV-based analysis. However, both analyses had poor negative predictive values for both *Enterobacteriaceae* and *Escherichia*/*Shigella* (all <23%). OTU-based analysis was slightly more accurate than ASV-based analysis, but neither performed remarkably well (all accuracy estimates <63%). Thus, with respect to *Enterobacteriaceae* and *Escherichia*/*Shigella*, 16S sequencing and culture results did not correlate well.

## Discussion

Few longitudinal studies have been conducted to identify short and long-lived members of the human gut microbiome, estimate rates of migration into and out of the gut ecosystem, or understand the functional impact of taxonomic turnover. Temporal variability is an important attribute of ecosystems affecting colonization dynamics [34]. In macrobiomes (e.g., animals), the decision to migrate or reside is a function of environmental condition-dependent fitness costs and benefits [34, 35]. It seems reasonable to hypothesize the same is true for human gut microbiome members and that evolutionary strategies leading to migration or residency result from realized fitness tradeoffs inside and outside the gut environment. Adaptation to one or both of these habitats, was previously proposed as an evolutionary strategy for *E. coli* [36], and as a model, seems applicable to most gut microbiome species. A primary goal of this study was to assemble a longitudinal collection of stool samples from healthy human adults and begin to examine gut microbiome residency.

Two previous longitudinal 16S rRNA sequencing studies of healthy human adults [6, 7] both considered two individuals for between 6 and 15 months. Thus, our study (*n* = 8 participants for an of ~17 months) more than doubles the size of current datasets. Other longitudinal 16S datasets have been published, but their comparatively short sampling periods (e.g., 3 months) [8], use of alternative sequencing methods [9, 11], evaluation of controlled diets [9] and/or living conditions [37, 38] make them somewhat difficult to compare. That said, these studies all report that the microbiome is “stable” over long periods, sometimes even predicting that the same bacteria reside for >50 years [11, 12]. Such statements are based on taxonomic levels well above the unit of natural selection (i.e. populations) making it difficult to know whether and to what extent this so-called stability is biologically relevant. An extreme example suggested that a core microbiome was maintained over the course of an entire adult lifetime [12]. Our results cast serious doubt on this level of stability. We and others [1, 3, 39–41] have shown that clonal *E. coli* populations in the gut are dynamic and turn over on the order of months to years. Indeed, clones observed in only two of our eight participants resided for the entire sampling period.

Our results do not address whether *E. coli* clone dynamics scale to the rest of the microbiome, but culture-based evidence suggests that it does. For example, Faith et al. cultured and genome sequenced resident (up to 1.3 years) “strains” of the *Bacteroidetes*, *Firmicutes*, *Proteobacteria*, and *Actinobacteria* phyla from longitudinally collected stool samples [11]. They defined the same “strains” as isolates with genome coverage score of >0.96, which from a population genetic perspective would not necessarily be considered clonal, so these results are very much consistent with *E. coli* resident turnover in our study. Another study cultured *Bacteroides* species from longitudinal stool samples of 15 adults and found that some individuals harbored different strains over the study period [4]. So, how important is strain-level differences to gut microbiome function? Considering that the first three completed genomes of *E. coli* shared <40% of their enzyme coding loci [5] and the fact that 16S cannot resolve this level of diversity, the potential for clonal turnover to change gut function—even when upper-level taxonomy is “stable”—is great.

Previous approaches to examine *E. coli* residency in human adults have been limited to some degree by both sampling depth (within samples) and breadth (between individuals). According to rare events calculation, the average of 84 isolates per sample that we achieved allowed for confident detection of representative clones at ~2% relative abundance (power = 0.8, expected event rate lambda = 0.02, critical tolerance limit = 1), which is by far the most comprehensive study to date. It is important to recognize, however, that our approach was meant to capture diversity and so while our identification of resident clones (Fig. 3) should be reasonably robust, estimates of relative abundance (Supplementary Fig. 13) may be somewhat biased. Regardless, our results provide clear evidence that *E. coli* was by far the most abundant and resident gut species of *Enterobacteriaceae* in at least this cohort, and that *E. coli* of the A, B2.3, and F phylogroups were more likely to reside over long periods of time compared to other lineages. Interestingly, phylogroup B2 may also be a common resident of infants [42]. A common observation from cross-sectional *E. coli* studies is that one clone is “dominant” in stool samples, or at a high relative abundance. One interpretation is that this clone represents the resident clone. Caution should be used when making this assumption as we observed dramatic shifts in phylogroup abundance through time (Supplementary Fig. 13). Future examination of seemingly adaptive associations between phylogenetically related clones and residence may provide a better understanding of the microbial factors underlying gut colonization and persistence. Whether the most important adaptations arose long ago as our results with *E. coli* suggest or whether they arose over much shorter time scales (as was recently suggested for *Bacteroides fragilis* [43]) requires more data from healthy participants.

If *E. coli* is so prevalent among humans, why is it often missing from gut microbiome datasets? A principle factor seems to be that the accuracy of 16S sequencing, regardless of analytical approach (OTU or ASV), is poor for *Enterobacteriaceae* and *Escherichia/Shigella*, which is consistent with analyses of simple mock communities (n ≤ 20 members) where *E. coli* cells or genomic DNA were mixed at relatively equal abundances with that of other gut bacteria [13, 44]. In addition, nearly all *Enterobacteriaceae* was *E. coli* in our cohort, which is not the case for other human gut microbiome families, such as the *Bacteroidaceae*, that can be comprised of at least 20 different species [4]. Thus, there seems to be ample evidence that 16S sequencing should not be used to estimate the absolute abundance of microbiome members nor should it be used to determine whether taxa are absent from a sample without first benchmarking a high correlation between these observations and the gold-standard of culture. This also means that 16S-based statements regarding the “dominance” of certain higher order taxa over others (e.g. *Bacteroidaceae* vs. *Enterobacteriaceae*) should be avoided, as they could mean very little at the individual population or species levels.

Regarding the use of OTU versus ASV-based analysis, we found these two approaches yielded nearly identical ordinations (a common proxy for community diversity) for five of eight participants but produced different results for the remaining three. This suggests that ASV-analysis differs in discriminatory power from sample-to-sample and that comparisons are sometimes (and perhaps unpredictably) made at different taxonomic levels (e.g., species vs. genera). In contrast, the 97% 16S OTU cut-off is a reasonable approximation of both the 95% average nucleotide identity of genomes and 70% DNA-DNA hybridization cut-offs that are commonly used to differentiate bacterial species [45, 46], thus ensuring that communities are compared equally with respect to taxonomic level. Also, it is unclear how variant 16S alleles (within genomes) contribute to ASV-based analyses, and whether, for example, ASVs that classify to the same species and are always observed together represent different alleles, populations, species, or higher taxa [47]. We showed that OTU-based analysis was the more accurate approach for detecting both *Enterobacteriaceae* and *Escherichia/Shigella*. That said, given the widespread use of both analyses, more diagnostic testing against the gold-standard of culture is needed to benchmark their biological relevance.

Although not the focus of this study, it is likely that both host and microbial factors are involved in microbiome residency. There is evidence from shorter-term longitudinal studies that host factors (e.g., antibiotic use [2] and travel [48]) are important as are socio-economic factors (e.g., phylogroup B2 *E. coli* were more prevalent among people from industrialized compared to developing countries [49, 50]). There is also evidence that GI distress (e.g., natural and induced diarrhea) does not necessarily purge resident strains from a host [51, 40]. Similarly, resident *E. coli* were remarkably stable in the face of enterotoxigenic *E. coli* infection and antibiotic treatment [52]. Microbial virulence factors [53] seem to be important in *E. coli* residency, and especially factors associated with uropathogenesis (e.g., adhesins, siderophores, and hemolysins [42, 53]). Consistent with this finding, clonotyping suggests that A and B2.3 residents in our study belong to sequence types commonly observed in urinary tract infections [21] (Supplementary Table 2). Microbial metabolism may also be important for residency [54, 55]. Freter’s nutrient niche hypothesis suggests that metabolism determines residency such that only clones that most efficiently utilize limiting nutrient(s) will persist [55, 56]. Finally, direct clone interference through bacteriocins or bacteriophage production may be important [40, 57]. Determining which of these factors predict residency will certainly be useful in microbiome-directed therapies.

Few studies have considered lactose non-fermenting (lac-) *E. coli*, but we found them to be quite common. For example, four long-term (>50 day) lac- resident clones belonging to three different phylogroups were observed among half (*n* = 4) of our cohort; lac- clones belonging to six of the nine observed phylogroups were identified; and lac- clones comprised 17% of the 120 *E. coli* clones observed. These results are not entirely without precedent as Sears et al. [51] and a published doctoral thesis [58] reported several lac- residents in healthy adults. Interestingly, lac- *E. coli* also appear to be associated with uropathogenesis [59, 60], which is consistent with the above mentioned overlap with virulence factors and gut residency. The combined evidence should compel future studies to consider all *E. coli*, regardless of lactose utilization.

## Supplementary information

Supplementary Material
