# Supplementary material for: Rethinking gut microbiome residency and the Enterobacteriaceae in healthy human adults
Source: ISME J. 2019 May 14;13(9):2306–18. doi: 10.1038/s41396-019-0435-7 (PMC6776003; doi:10.1038/s41396-019-0435-7)
Supplement: Supplementary file 1 — Supplementary Material [file 41396_2019_435_MOESM1_ESM.docx]

**Supplementary Figure 1.** Schematic of Clermont *et al.* multiplex PCR method for phylogrouping *E. coli* and cryptic *Escherichia* clade isolates. The only modification to the original protocol was that reaction volumes were cut in half and 1.6 µL of a 1:10 dilution overnight cultures (in sterile water) was used as template. Representative isolates belonging to AC, DE.1, ECC and were further differentiated as described by Clermont *et al.*


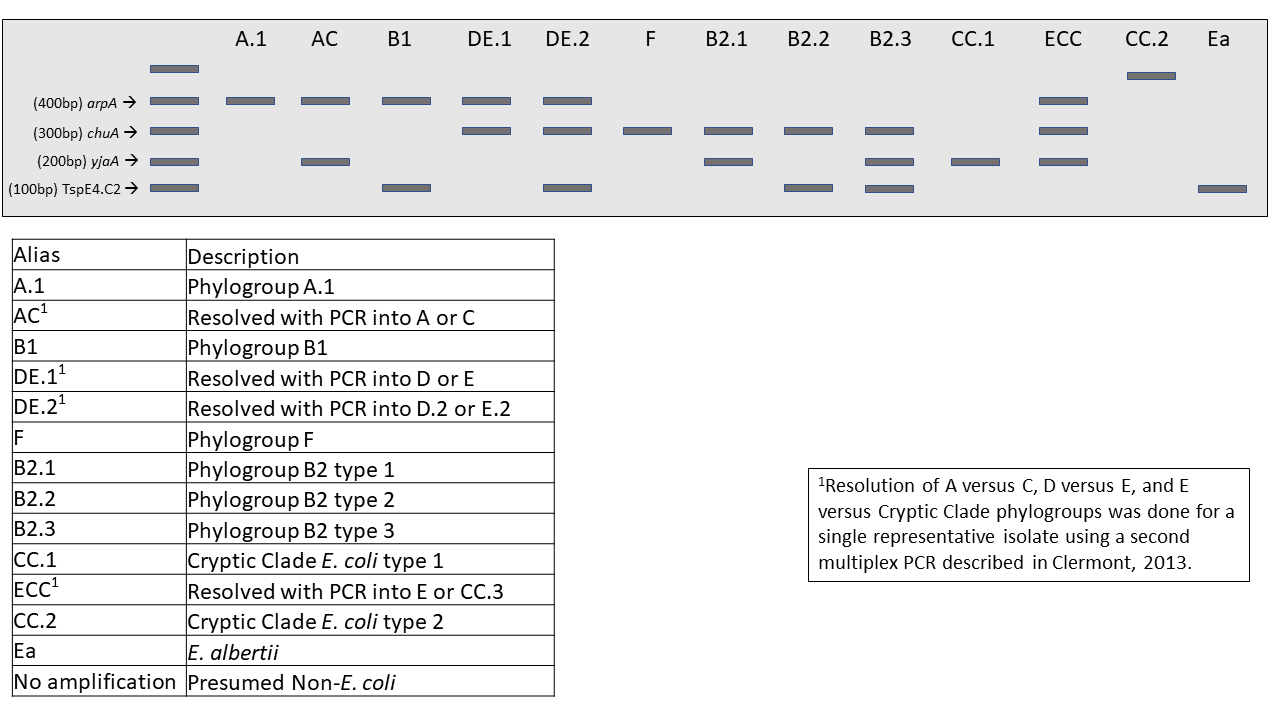


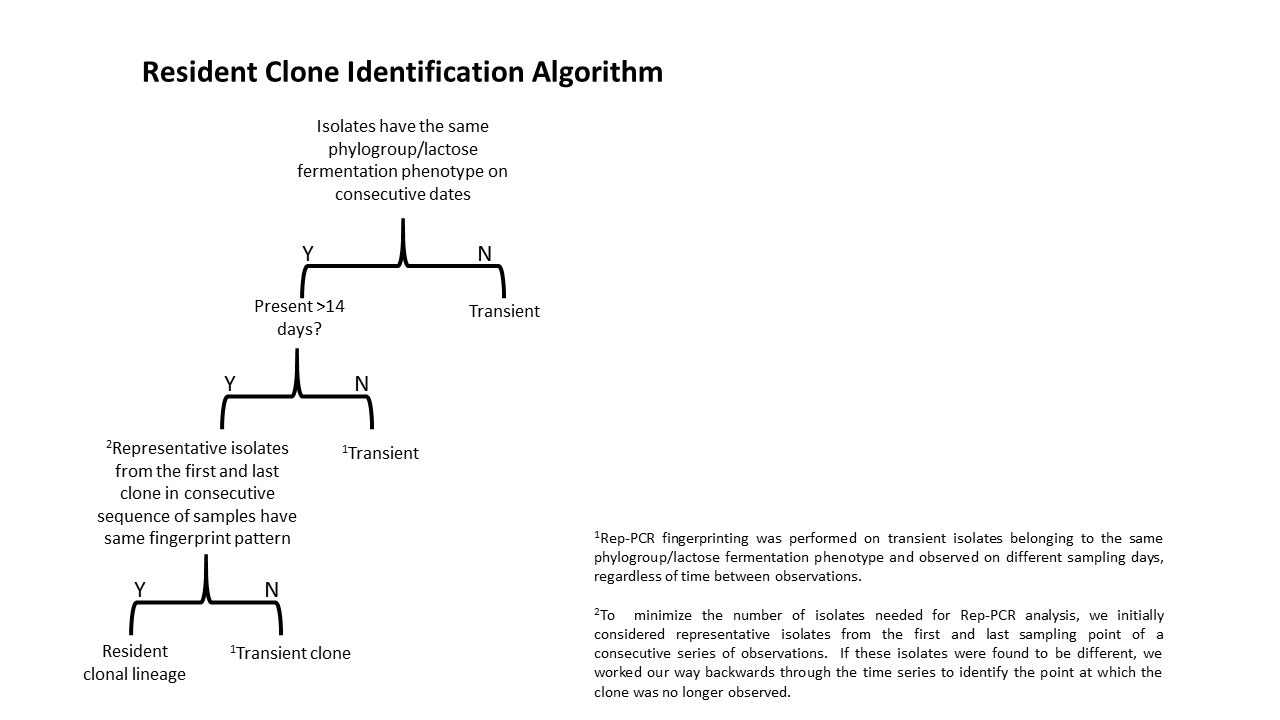
**Supplementary Figure 2**. Flow chart showing the algorithm used to identify resident and transient clones.

**Supplementary Figure 3.** Timeline of stool sample collection for eight participants. An average of 49 samples were collected per participant. Hashes denote individual samples plated onto MacConkey agar (population-level dynamics). Red dots were used for 16S rRNA sequence analysis (community-level dynamics).

**Supplementary Figure 4**. Silhouette plots of OTUs (left) and ASVs (right). ASV-analysis produced larger mean silhouette widths compared to OTUs (0.52 vs 0.44) and supported 8 clusters instead of the 7 clusters in OTU-based analysis.


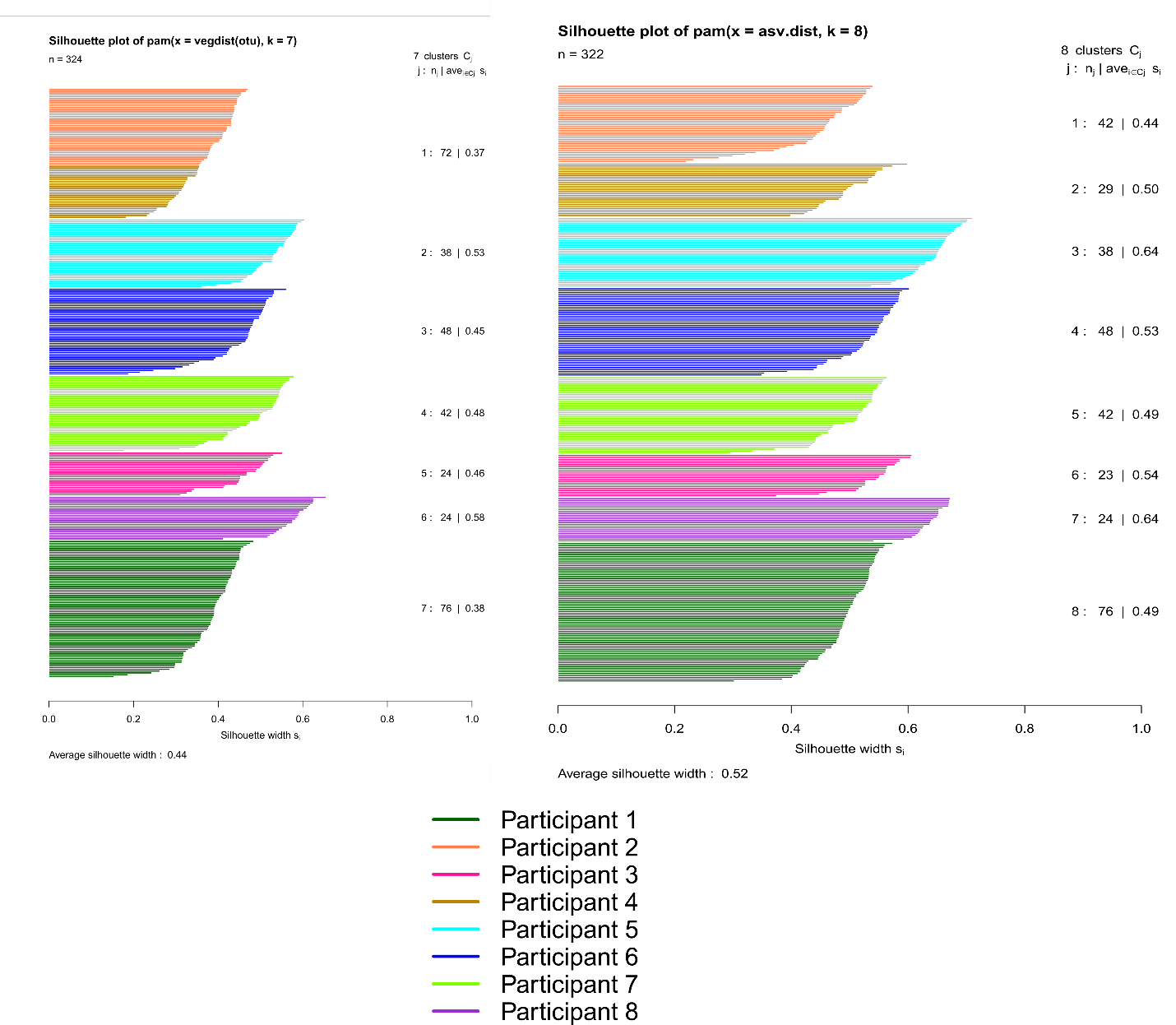


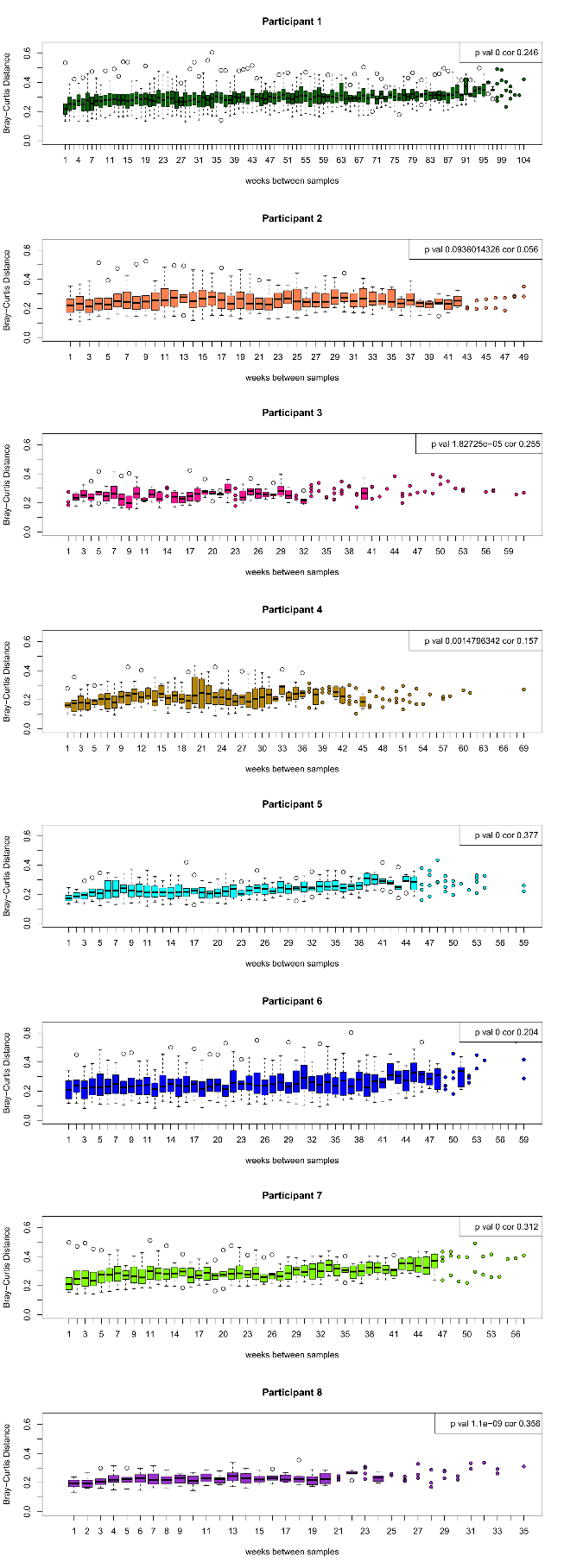
**Supplementary Figure 5.** Bray-Curtis dissimilarities for all pairwise comparisons between samples within participants with respect to time. Dissimilarities were categorized according to the length of time in weeks between samples (x-axis) and ranked from shortest (left) to the longest (right) time periods. Pearson correlation coefficients and p-values shown in upper right box of each plot. Dots are shown for times comprised of <5 comparisons, otherwise comparisons are summarized with box-and-whisker plots.

**Supplementary Figure 6.** 16S rRNA sequencing-based ASV analysis. Non-metric multidimensional scaling (NMDS) of samples colored according to participants and supported by PAM clustering.


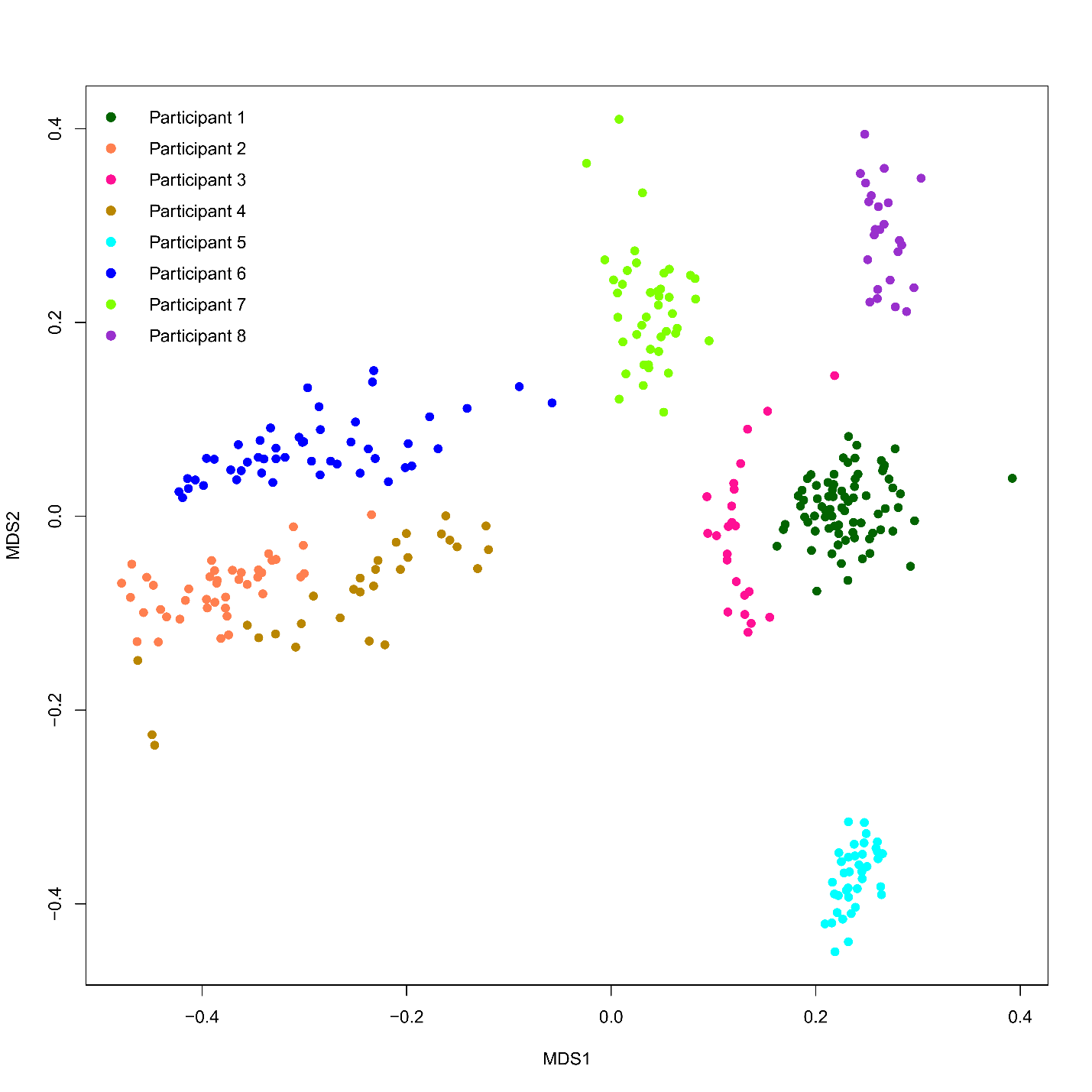


**Supplementary Figure 7.** Procrustes analysis of OTU- and ASV-based NMDS for each participant. The inset in each plot contains the sum of squared distances (SSDs).


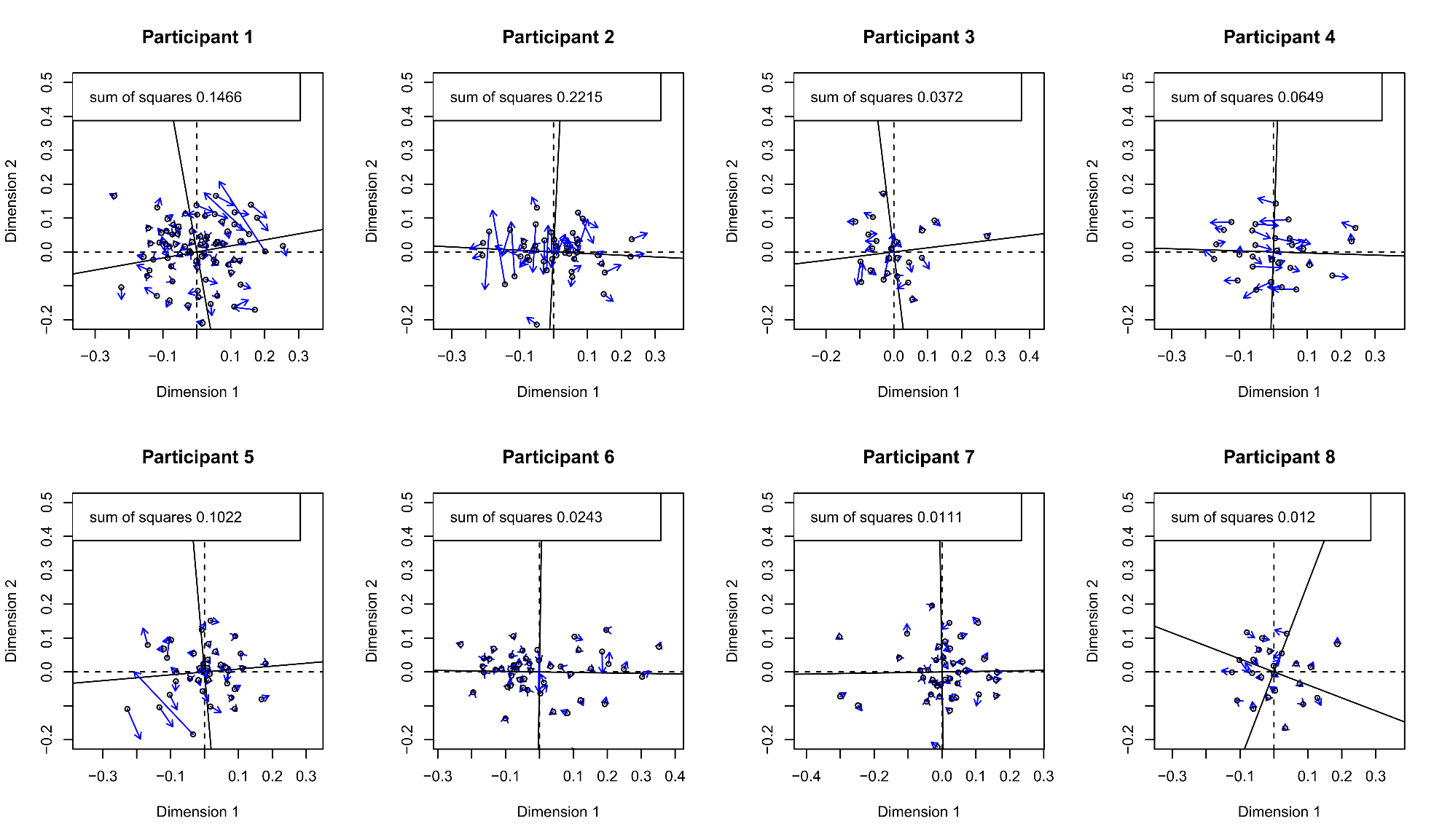


**Supplementary Figure 8.** Alpha diversity as Inverse Simpson’s estimates (Invsimp) based on OTUs (purple) and ASVs (forest green). Pearson correlation coefficients shown in each plot. All p-values < 0.00001.


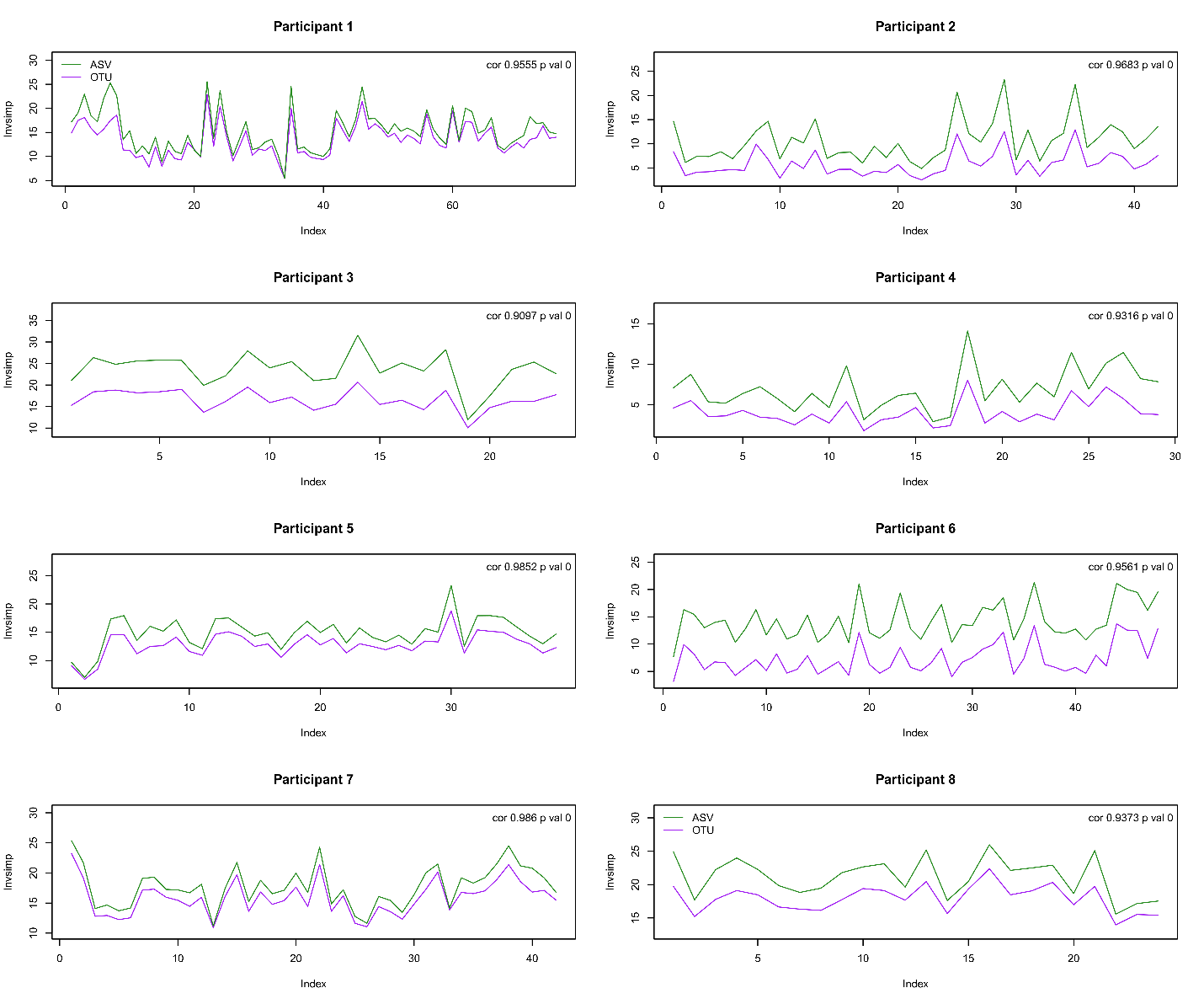


**Supplementary Figure 9.** Beta diversity (Bray-Curtis dissimilarity) estimates based on OTUs (purple) and ASVs (forest green). Pearson correlation coefficients shown in each plot. All p-values < 0.00001.


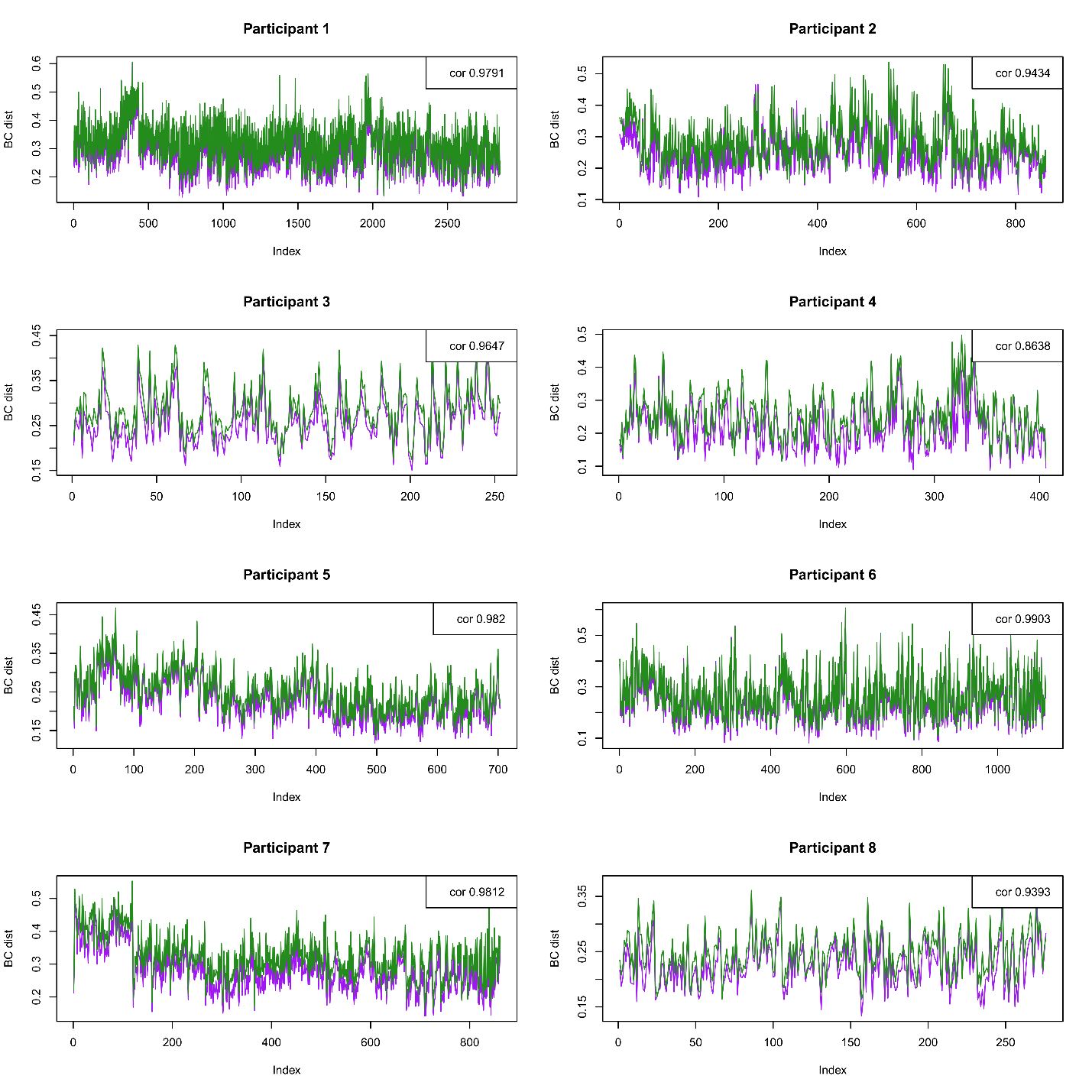


**Supplementary Figure 10.** Example of residency period estimation. Three different estimates of time were generated (Max, Ave, and Min), where the number of days between observations were estimated differently (e.g. Max = Date 4 – Date 1 versus Ave = Date 4 – Date 2). If the number of days between observations was greater than 14, then this would be considered a residency period. We also allowed for a certain amount of time between observations, or gaps, in each estimate. If gaps were ≤30 days (e.g. number of days between Date 4 and Date 5 for the Max estimate), then the residence period was assumed to continue (e.g. if the number of days between Dates 1 and 4 was greater than 14, and the number of days between Dates 4 and 5 was less than or equal to 30 days, then the residence period for the Max estimate was equal to the entire date range, or the number of days between Date 1 and 6).


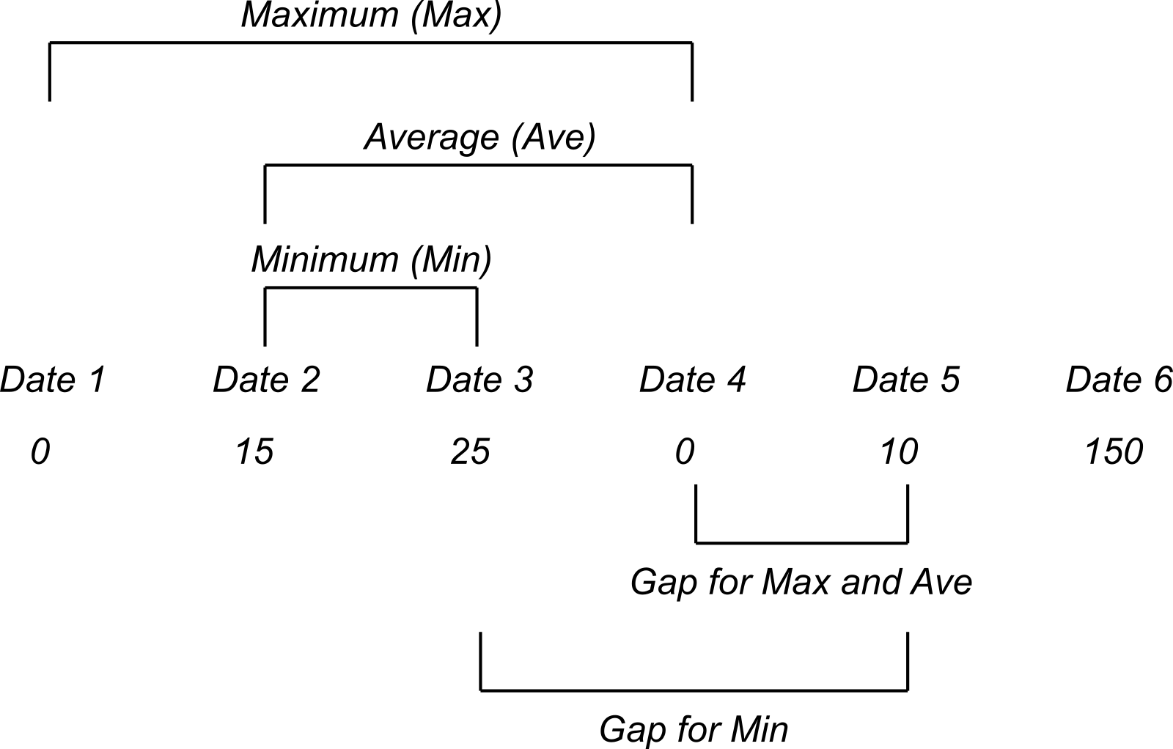


**Supplementary Figure 11.** A core microbiome does not exist between all participants. The number of “always resident” OTUs (see text) were plotted against the number of participants evaluated according to three operationally defined residence times (Minimum—Min, Average—Ave, and Maximum—Max). A non-linear regression model (one phase decay, unconstrained plateau) was fit to each line and best-fit values of model parameters are shown for R^2^ and lines plateaus. None of the plateaus were significantly different from 0 (Extra sum-of-squares F test; p > 0.05).


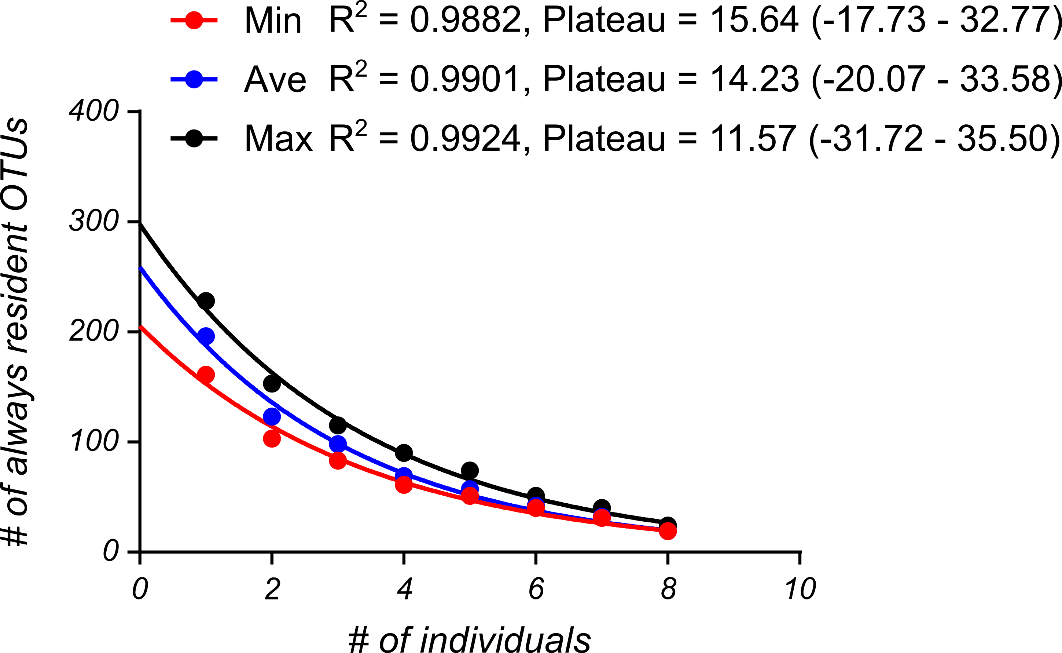


**Supplementary Figure 12.** Stacked bar charts of microbiome residency using the Min (top panels) and Max (bottom panels) estimates and for OTUs (left panels) and ASVs (right panels). The number of observed OTUs and ASVs are shown with respect to percentile rank according to the percentage of time present in each participant using the average residency estimate. Black stacks in each bar correspond to transient OTUs and ASVs (i.e. never resident).


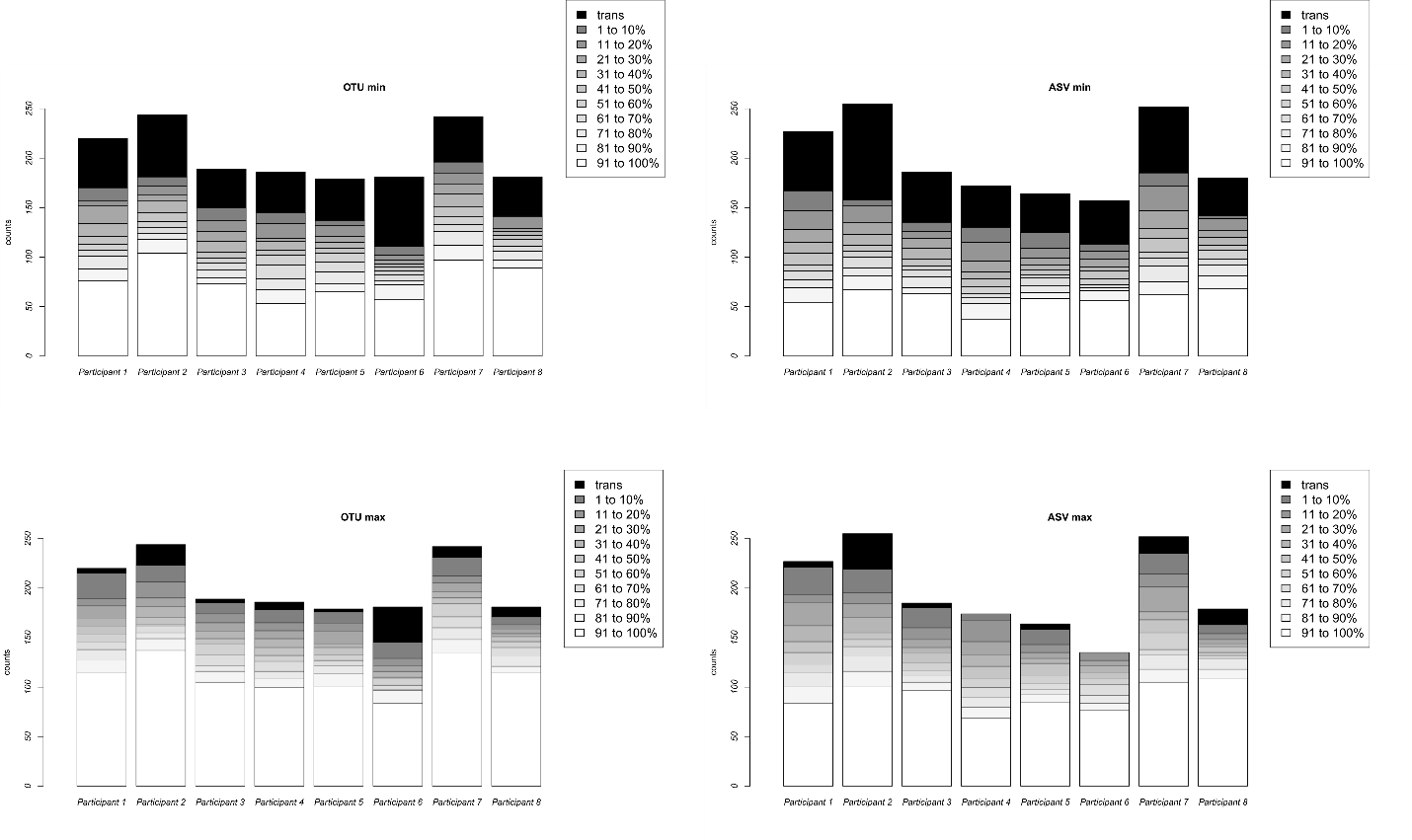


**Supplementary Figure 13.** Relative abundance of *E. coli* phylogroups and non-*E. coli* *Enterobacteriaceae* within participants through time. Note: the proportions of phylogroups and non-*E. coli* were skewed to capture the greatest amount of colony morphology diversity.


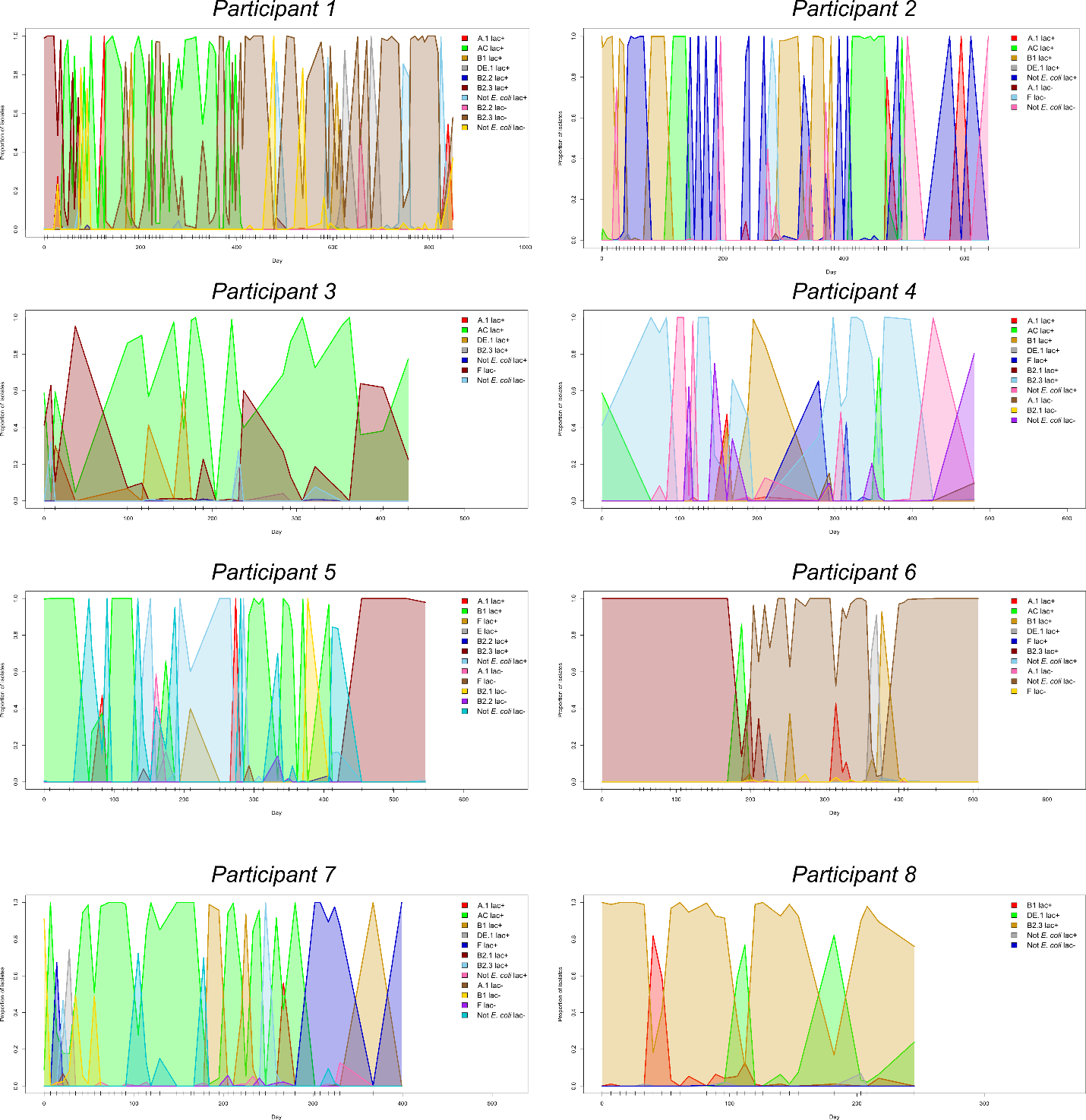


**Supplementary Figure 14.** All *Enterobacteriaceae* clones were plotted according to their log 10 transformed Max (A) and Min (B) residence time in days (A). Clones were grouped according their phylogroup (*E. coli*) or into an “Other” category if ≤6 representative isolates were observed (Panel A Other = B2.1, n=4; B2.2, n=4; D, n=6; E, n=4; Cryptic *Escherichia* clade IV, n=1; *C. freundii*, n=1; *K. pneumoniae*, n=1; Panel B Other = A.1, n= 3; D, n=4; E, n=1; F, n=5; Cryptic *Escherichia* clade IV, n=1; *C. freundii*, n=1; *K. pneumoniae*, n=1). Groups had significantly different residence time using the Max estimate (ANOVA, F = 3.776, p = 0.0034) and following correction for multiple comparisons (Holm-Sidak’s test), groups A, B2.3, and F resided significantly longer than A.1 (p = 0.0388, p = 0.0127, 0.0406 respectively). The overall ANOVA using the Min estimate neared but did not reach significance (F = 2.46, p = 0.0735).


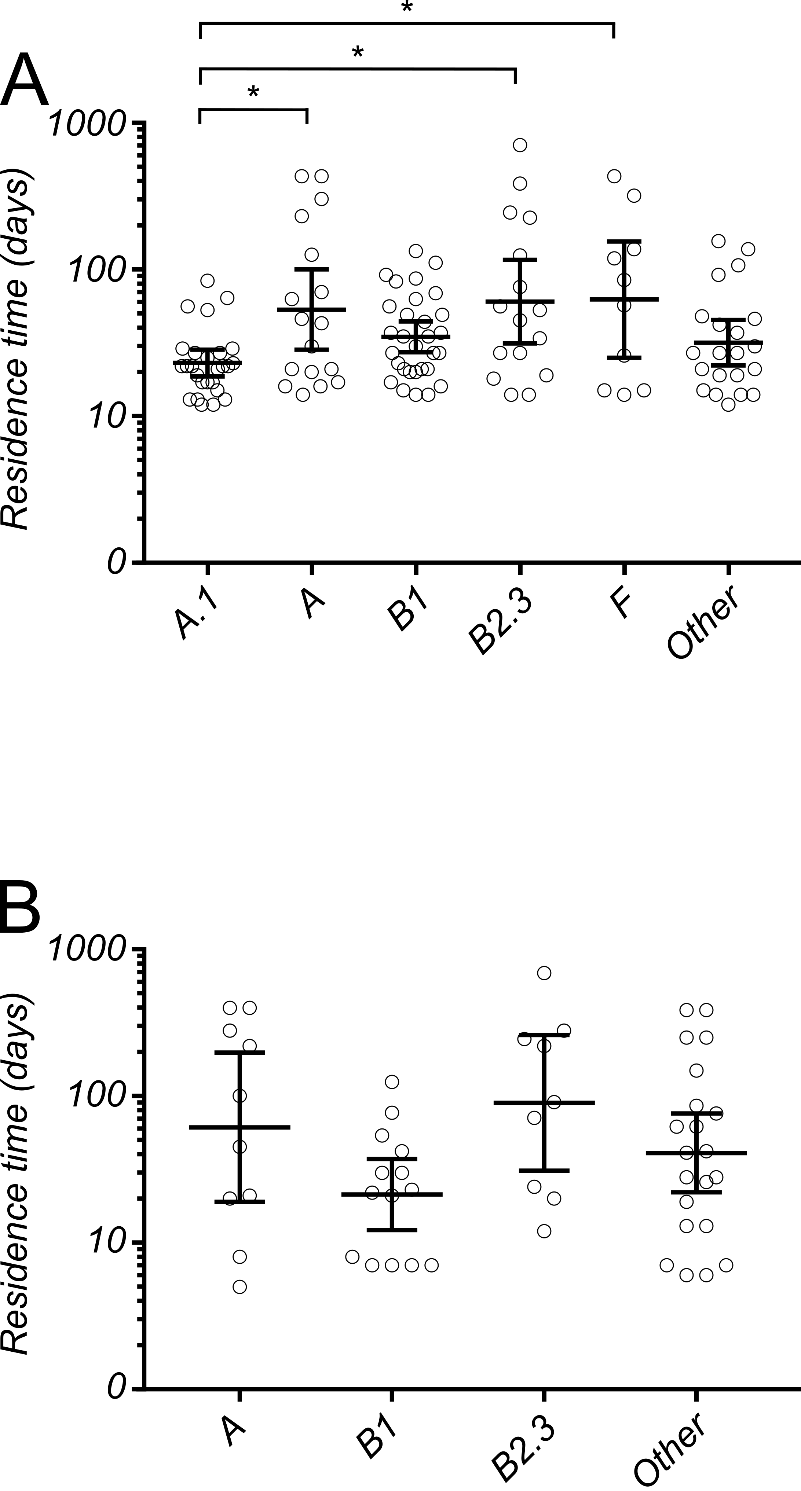


|  | Participant 1 | Participant 2 | Participant 3 | Participant 4 | Participant 5 | Participant 6 | Participant 7 | Participant 8 |
| --- | --- | --- | --- | --- | --- | --- | --- | --- |
| # of non-*E . coli* isolates | 768 | 1750 | 78 | 564 | 888 | 32 | 227 | 7 |
| # of potential non-*E. coli* resident isolates | 629 | 770 | 77 | 534 | 888 | 32 | 227 | 7 |
| # of non-*E. coli* isolates screened | 192 | 191 | 77 | 132 | 192 | 32 | 144 | 7 |
| % of potential resident isolates screened | 31% | 25% | 100% | 25% | 22% | 100% | 63% | 100% |
| % of total non-*E. coli* isolates screened | 25% | 11% | 99% | 23% | 22% | 100% | 63% | 100% |
| non-*E. coli* species identified by API 20E test strips -- *resident | **Citrobacter freundii* | *Citrobacter youngae* | *Hafnia alvei* | *Citrobacter freundii* | *Citrobacter freundii* |  | *Klebsiella oxytoca* |  |
|  | *Enterobacter cloacae* | *Cronobacter spp* |  | *Citrobacter koseri* | *Citrobacter koseri* |  | *Citrobacter freundii* |  |
|  | *Hafnia alvei* | *Cryptic Clade IV *Escherichia coli* |  | *Enterobacter cloacae* | *Citrobacter youngae* |  |  |  |
|  | *Klebsiella pneumoniae* | *Enterobacter amnigenus* |  | *Klebsiella oxytoca* | *Enterobacter cloacae* |  |  |  |
|  |  | *Enterobacter cloacae* |  | *Klebsiella pneumoniae* | *Klebsiella oxytoca* |  |  |  |
|  |  | *Escherichia hermannii* |  | *Klyvera spp* | **Klebsiella pneumoniae* |  |  |  |
|  |  | *Hafnia alvei* |  |  | *Kluyvera spp* |  |  |  |
|  |  | *Klebsiella oxytoca* |  |  | *Pseudomonas aeruginosa* |  |  |  |
|  |  | *Klebsiella pneumoniae* |  |  | *Serratia fonticola* |  |  |  |
|  |  |  |  |  |  |  |  |  |
|  |  |  |  |  |  |  |  |  |

**Supplementary Table 1.** Summary of non-*E. coli Enterobacteriaceae* characterization and identification by API 20E biochemical profiling.

**Supplementary Table 2.** Clonotyping results for representative isolates of resident clones belonging to phylogroups A and B2.3.

| Phylogroup | Lactose Fermentation | Avg Days resident | Participant ID | Clone Number | Septatyping result | ST (fimH allele) |
| --- | --- | --- | --- | --- | --- | --- |
| A | lac+ | 118 | Participant 1 | Clone 3 | 750 | new |
| A | lac+ | 28 | Participant 1 | Clone 7 | 771 | ST10 (fimH23/27) |
| A | lac+ | 224 | Participant 1 | Clone 10 | 771 | ST10 (fimH23/27) |
| B2.3 | lac+ | 76 | Participant 1 | Clone 1 | 530 | ST127 (fimH2) |
| B2.3 | lac+ | 34 | Participant 1 | Clone 21 | NA | NA* |
| B2.3 | lac- | 689 | Participant 1 | Clone 8 | 551 | ST144 (fimH54) |
| A | lac+ | 12 | Participant 2 | Clone 13 | 761 | ST10 (fimH30/31) |
| A | lac+ | 63 | Participant 2 | Clone 14 | 771 | ST10 (fimH23/27) |
| A | lac+ | 28 | Participant 2 | Clone 6 | 771 | ST10 (fimH23/27) |
| A | lac+ | 16 | Participant 2 | Clone 2 | 750 | new |
| A | lac+ | 433 | Participant 3 | Clone 1 | 761 | ST10 (fimH30/31) |
| A | lac+ | 433 | Participant 3 | Clone 2 | 761 | ST10 (fimH30/31) |
| B2.3 | lac+ | 316 | Participant 4 | Clone 1 | 620 | ST73 (fimH9/10) |
| B2.3 | lac+ | 27 | Participant 4 | Clone 6 | 520 | ST372 (fimH9/12) |
| B2.3 | lac+ | 91 | Participant 5 | Clone 18 | 530 | ST127 (fimH2) |
| B2.3 | lac+ | 226 | Participant 6 | Clone 1 | 721 | ST95 (fimH15) |
| A | lac+ | 302 | Participant 7 | Clone 1 | 751 | ST10 (fimH54) |
| B2.3 | lac+ | 20 | Participant 7 | Clone 12 | 510 | ST131 (fimH22) |
| B2.3 | lac+ | 245 | Participant 8 | Clone 1 | 721 | ST95 (fimH15) |
| *No PCR result obtained for this isolate |  |  |  |  |  |  |

**Supplementary Table 3.** Estimated diagnostics for ASV- and OTU-based analyses compared to culture-based results for Escherichia and Enterobacteriaceae.

| ASV-*Escherichia* | Count |  |  |
| --- | --- | --- | --- |
| True negative | 35 |  |  |
| False negative | 161 |  |  |
| True positive | 126 |  |  |
| False positive | 0 |  |  |
|  | Estimate | Lower 95% CL | Upper 95% CL |
| Sensitivity | 43.90 | 38.08 | 49.86 |
| Specificity | 100.00 | 90.00 | 100.00 |
| Positive Predictive Value | 100.00 |  |  |
| Negative Predictive Value | 17.86 | 16.40 | 19.41 |
| Accuracy | 50.00 | 44.41 | 55.59 |
|  |  |  |  |
| ASV-*Enterobacteriaceae* |  |  |  |
| True negative | 16 |  |  |
| False negative | 173 |  |  |
| True positive | 133 |  |  |
| False positive | 0 |  |  |
|  | Estimate | Lower 95% CL | Upper 95% CL |
| Sensitivity | 43.46 | 37.83 | 49.22 |
| Specificity | 100.00 | 79.40 | 100.00 |
| Positive Predictive Value | 100.00 |  |  |
| Negative Predictive Value | 8.47 | 7.73 | 9.26 |
| Accuracy | 46.27 | 40.73 | 51.89 |
|  |  |  |  |
| OTU-*Escherichia* | Count |  |  |
| True negative | 35 |  |  |
| False negative | 120 |  |  |
| True positive | 169 |  |  |
| False positive | 0 |  |  |
|  | Estimate | Lower 95% CL | Upper 95% CL |
| Sensitivity | 58.48 | 52.56 | 64.22 |
| Specificity | 100.00 | 90.00 | 100.00 |
| Positive Predictive Value | 100.00 |  |  |
| Negative Predictive Value | 22.58 | 20.28 | 25.06 |
| Accuracy | 62.96 | 57.45 | 68.24 |
|  |  |  |  |
| OTU-*Enterobacteriaceae* | Count |  |  |
| True negative | 16 |  |  |
| False negative | 23 |  |  |
| True positive | 185 |  |  |
| False positive | 0 |  |  |
|  | Estimate | Lower 95% CL | Upper 95% CL |
| Sensitivity | 60.06 | 54.36 | 65.58 |
| Specificity | 100.00 | 79.41 | 100.00 |
| Positive Predictive Value | 100.00 |  |  |
| Negative Predictive Value | 11.51 | 10.19 | 12.98 |
| Accuracy | 62.04 | 56.51 | 67.34 |
